# Enhancing monolignol ferulate conjugate levels in poplar lignin via *OsFMT1*

**DOI:** 10.1186/s13068-024-02544-y

**Published:** 2024-07-13

**Authors:** Faride Unda, Lisanne de Vries, Steven D. Karlen, Jordan Rainbow, Chengcheng Zhang, Laura E. Bartley, Hoon Kim, John Ralph, Shawn D. Mansfield

**Affiliations:** 1 Department of Wood Science, Faculty of Forestry, University of British Columbia, Vancouver, BC V6T 1Z4 Canada; 2 US Department of Energy (DOE) Great Lakes Bioenergy Research Center, The Wisconsin Energy Institute, University of Wisconsin-Madison, Madison, WI 53726 USA; 3 Department of Biochemistry, University of Wisconsin-Madison, Madison, WI 53706 USA; 4 Institute of Biological Chemistry, Washington State University, Pullman, WA 99164 USA; 5 Department of Microbiology and Plant Biology, University of Oklahoma, Norman, OK 73019 USA; 6 US Department of Agriculture (USDA), Forest Service, Forest Products Laboratory (FPL), Madison, WI 53726 USA; 7 Botany Department, Faculty of Science, University of British Columbia, Vancouver, BC V6T 1Z4 Canada

**Keywords:** Lignification, Phenylpropanoid biosynthetic pathway, Feruloyl-CoA monolignol transferase, Biomass, UV–Vis spectroscopy, Cell wall characterization

## Abstract

**Background:**

The phenolic polymer lignin is one of the primary chemical constituents of the plant secondary cell wall. Due to the inherent plasticity of lignin biosynthesis, several phenolic monomers have been shown to be incorporated into the polymer, as long as the monomer can undergo radicalization so it can participate in coupling reactions. In this study, we significantly enhance the level of incorporation of monolignol ferulate conjugates into the lignin polymer to improve the digestibility of lignocellulosic biomass.

**Results:**

Overexpression of a rice Feruloyl-CoA Monolignol Transferase (*FMT*), *OsFMT1*, in hybrid poplar (*Populus alba* x *grandidentata*) produced transgenic trees clearly displaying increased cell wall-bound ester-linked ferulate, *p-*hydroxybenzoate, and *p-*coumarate, all of which are in the lignin cell wall fraction, as shown by NMR and DFRC. We also demonstrate the use of a novel UV–Vis spectroscopic technique to rapidly screen plants for the presence of both ferulate and *p-*hydroxybenzoate esters. Lastly we show, via saccharification assays, that the *OsFMT1* transgenic p oplars have significantly improved processing efficiency compared to wild-type and *Angelica sinensis*-*FMT-expressing* poplars.

**Conclusions:**

The findings demonstrate that *OsFMT1* has a broad substrate specificity and a higher catalytic efficiency compared to the previously published FMT from *Angelica sinensis* (*AsFMT*). Importantly, enhanced wood processability makes *OsFMT1* a promising gene to optimize the composition of lignocellulosic biomass.

**Supplementary Information:**

The online version contains supplementary material available at 10.1186/s13068-024-02544-y.

## Background

A primary chemical constituent of the plant cell wall is lignin, a phenolic polymer found in many cell types that provides structural support, facilitates water transport, and forms a barrier against pathogens. The canonical monolignols *p*-coumaryl, coniferyl, and sinapyl alcohols are the monomeric building blocks of the lignin polymer. Following synthesis in the cytoplasm by the phenylpropanoid biosynthetic pathway, the monolignols are exported to the cell wall where they undergo radicalization, catalyzed by laccases and peroxidases [1–3]. Once incorporated into lignin, these canonical monolignols produce *p*-hydroxyphenyl (H), guaiacyl (G), and syringyl (S) units. Given the inherent combinatorial coupling of radicals to generate the polymer, lignin is a highly metabolically malleable racemic polymer that varies between species, cell types, and developmental stages. Numerous studies have shown that, along with the canonical monolignols, non-canonical phenolic monomers can be incorporated into the lignin polymer as long as they can undergo radicalization [4–6]. These non-canonical monomers are naturally occurring, but can also be ectopically engineered to increase processing efficiency of lignocellulosic biomass and lignin’s inherent value [7]. Examples of non-canonical monomers are monolignols acylated by acetate, benzoate, *p*-hydroxybenzoate (*p*HBA), *p*-coumarate (*p*CA), and ferulate (FA) [8–17], flavonoids [18–20], hydroxystilbenes [21, 22], other phenolates such as protocatechuate [23, 24], scopoletin (a coumarin) [25], and curcumin (a diarylheptanoid) [26].

Initial attempts to engineer poplar with monolignol-acylating enzymes produced significant, if arguably modest, effects (e.g., cell-wall bound *p*HBA was increased from 5.8% in wild-type trees to 7.9% in C4H::*p*HBMT1 line [8]). We hypothesized that, in contrast to *p*CA and *p*HBA moieties that prefer radical transfer over radical coupling, the more electron-rich and reactive ferulate moiety would undergo radical coupling during lignification [27, 28]. Monolignol ferulate ester conjugates (ML-FA) might thus be incorporated into the main chain of the lignin polymer creating so-called “Zip-lignin”. Such lignin is more readily degradable under mild alkaline conditions due to the presence of chemically labile ester bonds in the lignin polymer chains. A ferulate monolignol transferase (FMT) from *Angelica sinensis*, *AsFMT*, was the first to be transformed into poplar under the control of a secondary cell wall cellulose synthase promoter [10, 29–31]. That FMT enzyme belongs to class III of the so-called “BAHD” acyltransferase family and can form monolignol ferulate esters from feruloyl-CoA and a canonical monolignol [10]. By expressing *AsFMT* in poplar, Wilkerson et al. [10] concluded ML-FA conjugates were incorporated into the poplar lignin, conferring improved saccharification efficiency (i.e., the amount of polymeric carbohydrate that can be converted to monomers) under mild alkaline conditions (6.25 mM NaOH, 90 °C, 3 h), even though there was no impact on overall plant agronomics [10]. Woody tissue from these same plants showed improved pulping efficiency [29] and better processing efficiency when subjected to additional pretreatment regimes [30, 31].

A second BAHD-type FMT was identified in an activation-tagged rice mutant (*OsAT5-D1*) that showed an increase in the cell wall ferulate content compared to wild-type (WT) plants [32]. To confirm that the OsAT5 had an FMT activity, transgenic rice lines overexpressing the *OsAT5* (hereafter called *OsFMT1*) gene driven by the *ZmUbi* promoter were shown to increase the level of monolignol dihydroferulate (ML-DHFA) conjugates via derivatization followed by reductive cleavage (DFRC), confirming native FMT activity and the incorporation of the resulting FA conjugates into lignin [33]. DFRC is an analytical method for cleaving the predominant β-aryl ether linkages in lignin while retaining the esters, releasing diagnostic (dihydro, acetylated) monolignol conjugates in addition to the usual monolignols [33–35].

To attempt to extend the limits of ML-FA incorporation into poplar xylem compared to WT, we compared the two previously identified FMTs in the same plant species. We overexpressed *OsFMT1* driven by the *Arabidopsis thaliana (At) C4H* promoter in hybrid poplar (*Populus alba x P. grandidentata*). As *C4H* is a core phenylpropanoid gene, *OsFMT1* should be expressed during the biosynthesis of monolignols, giving ample substrate for the synthesis of the ML-FA conjugates. We characterized the transformed *OsFMT1* tree samples by NMR, UV–Vis analysis, alkaline hydrolysis, enzymatic digestion, and microscopy. We confirmed that *OsFMT1* has a broader substrate specificity than *As*FMT, as we observed an increase in ferulate (FA), *p*HBA, and *p*CA incorporation. Furthermore, the *OsFMT1* poplars driven by the *AtC4H* promoter displayed significantly increased ML-FA levels compared to the original *AsFMT* poplars.

## Methods

Methodology describing the generation, selection, and cultivation of transgenic hybrid poplar trees and autofluorescence microscopy is included in the Supporting Information (Methods S1).

### Cell wall composition

Stem samples from greenhouse-grown trees were subjected to acid hydrolysis using a modified Klason lignin method [36, 37]. The method is described in detail by Unda et al. [24].

The lignin monomer composition was determined following a modified thioacidolysis procedure [38]. Quantification via the procedure is described by Unda et al. [24].

### Cell-wall-bound acetyl groups and phenolics

Cell-wall-bound acetyl groups and phenolics were liberated by adding 1 mL of 2 M NaOH to approximately 30 mg of extractive-free ground xylem tissue along with 10 µL of *o*-anisic acid (10 mg mL^−1^) in screw-capped vials that were incubated at 30 °C for 24 h in a thermomixer set to 500 rpm. The mixture was acidified by adding 100 µL of 72% sulphuric acid and allowed to cool on ice. The vials were centrifuged at 13,000 rpm for 2 min, and the supernatants were filtered through 0.45-µm syringe filters. Quantification of acetic acid is described in detail by Unda et al. [24].

### Preparations of the extract-free cell-walls and enzymatically isolated lignin (EL)

Debarked dry poplar xylem tissue flour was solvent extracted with water (3 × 40 mL), 160 proof ethanol (3 × 40 mL), and acetone (1 × 40 mL). The extract free cell-walls were dried under high vacuum (15 mTorr) and used in the DFRC analysis. A fraction of the extract-free biomass (750 mg) was subjected to plenary ball-milling using a Fritsch Pulverisette 7 premium line mill with 20 mL agate grinding jars and 10 × 10 agate ball-bearings. The milling was performed at 600 rpm for 35 cycles of 10 min milling and 5 min resting to dissipate heat. The finely milled biomass was transferred to 50 mL falcon tubes using 25.5 mM sodium acetate buffer pH 5. The volume was adjusted to 45 mL, ~ 20 mg of Cellulysin (Calbiochem) was added, and the sample was incubated for 48 h on a shaker table at 30 °C shaking at 250 rpm. The digested sample was pelleted (Sorval biofuge primo; 8500 rpm/10,016 × *g* for 10 min) and subjected to a second treatment of Cellulysin digestion. After 48 h of further digestion the sample was again pelleted, washed three times with RO water, and freeze-dried to give enzyme lignin. The enzyme lignin was used in UV–Vis, NMR, and GPC analysis without further purification.

### Derivatization followed by reductive cleavage (DFRC)

DFRC was performed as previously described [34]. Briefly, 50 mg of extract-free whole-cell-wall material was subjected to the analysis. After zinc reduction and before solvent extraction of the product mixture, each sample was spiked with an internal standard (ISTD): 537 μg d_8_-G, 557 μg d_8_-S, 154 μg d_8_-S-*p*HBA, 100 μg d_10_-S-DD*p*CA and 53 μg d_10_-S-DDFA; the dx isotopologues derive from standards bearing d_3_-acetate groups and, in the case of the *p*CA and FA conjugates, hydrogenation with D_2_; the monolignols were g-D_2_ analogs that, after deuteroacetylation, are also d_8_ isotopologues of the biomass-released DFRC monomers [34]. Quantified DFRC products were diacetylated *cis*- and *trans*-isomers of *p*-coumaryl alcohol (H), coniferyl alcohol (G), sinapyl alcohol (S), coniferyl *p*-hydroxybenzoate (G-*p*HBA), sinapyl *p*-hydroxybenzoate (S-*p*HBA), coniferyl 7,8-dihydro-*p*-coumarate (G-DH*p*CA), sinapyl 7,8-dihydro-*p*-coumarate (S-DH*p*CA), coniferyl 7,8-dihydro-ferulate (G-DHFA), and sinapyl 7,8-dihydro-ferulate (S-DHFA) were detected using multiple reaction monitoring (MRM) mass detection and quantified by a linear 8-point calibration curve using area ratios vs. concentration ratios of analyte-to-ISTD. The ISTD parings were: d_8_-G for H and G; d_8_-S for S; d_8_-S-*p*HBA for G-*p*HBA and S-*p*HBA; d_10_-S-DD*p*CA for G-DH*p*CA, S-DH*p*CA, and G-DHFA; and d_10_-S-DDFA for S-DHFA. Table 1 provides the product distribution of the quantified and detected DFRC products. Supplemental Table S2 shows the mole% of H + G + S monolignols (including ML-conjugates) and the mole% of quantified monomers that represent each class of ML-conjugate (combining the G-conjugate and S-conjugate).

**Table 1:** Derivatization followed by reductive cleavage (DFRC) of *OsFMT* poplar transgenic lines and WT trees

	H_OH_ mol%	G_OH/OR_ mol%	S_OH/OR_ mol%	ML-*p*CA mol%	ML-FA mol%	ML-*p*HBA mol%
Line 4	0.2% ± < 0.1%	**39.0% ± 0.3%**	60.8% ± 0.3%	0.2% ± < 0.1%	**21.0% ± 0.6%**	3.8% ± 0.2%
Line 6	0.2% ± < 0.1%	**40.0% ± 2.1%**	**59.8% ± 2.1%**	0.3% ± < 0.1%	**23.3% ± 3.3%**	5.4% ± 1.2%
Line 10	0.2% ± < 0.1%	**42.0% ± 2.6%**	**57.8% ± 2.6%**	0.2% ± < 0.1%	**24.2% ± 3.9%**	5.0% ± 0.3%
Line 9	0.2% ± < 0.1%	**39.1% ± 0.9%**	**60.7% ± 0.9%**	0.2% ± < 0.1%	**21.3% ± 1.3%**	3.6% ± 0.3%
Line 7	0.2% ± < 0.1%	**38.2% ± 0.8%**	**61.6% ± 0.8%**	0.2% ± < 0.1%	**17.5% ± 1.8%**	3.0% ± 0.4%
Line 3	0.2% ± < 0.1%	27.1% ± 0.6%	72.6% ± 0.6%	0.1% ± < 0.1%	4.2% ± 0.5%	3.3% ± 0.4%
WT	0.2% ± < 0.1%	26.0% ± 0.3%	73.9% ± 0.3%	ND	2.1% ± 0.1%	2.7% ± 0.3%

Product ratio for quantified DFRC monomers, where Σ(quantified products) = 100%, H_OH_ = H_OH_; G_OH/OR_ = G_OH_ + G_FA_; S_OH/OR_ = S_OH_ + S_*p*CA_ + S_FA_ + S_*p*HBA_; Values are the average of *n* = 3 biological replicates ran in duplicate, with the standard error of the mean (SEM). ND = not detected. Bold values correspond to a statistical difference, determined via ANOVA and Dunnett’s post hoc test, *P* < 0.05. No significant differences were calculated for ML-*p*CA, as ML-*p*CA was not detected in WT

### 2D HSQC NMR analysis of the lignin

NMR samples were prepared by dissolving 15 mg of enzyme lignin in 0.5 mL of 4:1 (v/v) dimethyl sulfoxide-d_6_ (DMSO-d_6_): pyridine-d_5_. Two-dimensional ^1^H–^13^C HSQC NMR spectra were collected on a cryoprobe-equipped NEO 700 MHz spectrometer (Bruker Corp., Billerica, MA, USA) with an adiabatic-pulse program (hsqcetgpsisp2.2). Peak identifications were based on previous reports [39]. The spectra were calibrated on the dimethyl sulfoxide solvent peak at *δ*_H_ = 2.49 ppm and *δ*_C_ = 39.5 ppm, and volume integrations of the contour peaks were performed using TopSpin 4.08 (Bruker Corp.). Aromatic subunit proportions were expressed on an (S + G) basis where (S + G) = [G_2_ + ½(S_2/6_ + *S*'_2/6_)] = 100%, % *p*HBA = ½*p*HBA_2/6_/(S + G), %*p*CA = ½*p*CA_2/6_/(S + G), %FA = FA_6_/(S + G). Lignin side-chain unit proportions are expressed on an [*A*_*α*_ + *B*_*α*_ + *C*_*α*_ + *C'*_*α*_ = 100%] basis. The lignin subunit ratio is provided in (Table 2).

**Table 2:** Cell wall chemical composition of *OsFMT* transgenic lines and WT trees

	Arabinose	Rhamnose	Galactose	Glucose	Xylose	Mannose	Lignin (acid-insoluble)	Lignin (acid-soluble)	S/G
Line 4	0.43 ± 0.02	0.53 ± 0.01	0.94 ± 0.02	46.94 ± 0.75	20.15 ± 0.04	**1.79 ± 0.13**	**14.93 ± 0.12**	**2.57 ± 0.12**	2.84 ± 0.09
Line 6	0.45 ± 0.02	0.53 ± 0.01	0.89 ± 0.01	46.27 ± 0.34	20.23 ± 0.07	**1.89 ± 0.03**	16.11 ± 0.12	**2.74 ± 0.04**	**3.03 ± 0.07**
Line 10	0.46 < 0.01	0.54 ± 0.01	0.94 ± 0.03	47.31 ± 0.52	20.30 ± 0.28	2.41 ± 0.07	15.63 ± 0.24	**2.78 ± 0.05**	**3.01 ± 0.05**
Line 9	0.44 ± 0.02	**0.57 ± 0.01**	**1.00 ± 0.04**	46.81 ± 0.78	20.26 ± 0.50	**2.02 ± 0.06**	16.07 ± 0.20	**2.75 ± 0.06**	**3.04 ± 0.25**
Line 7	0.47 ± 0.01	0.50 ± 0.01	0.95 ± 0.02	48.58 ± 0.51	19.58 ± 0.15	**1.81 ± 0.02**	16.09 ± 0.23	**2.70 ± 0.02**	**2.95 ± 0.09**
Line 3	0.43 ± 0.02	0.46 ± 0.01	0.89 ± 0.03	46.57 ± 0.72	19.79 ± 0.22	**1.39 ± 0.03**	15.98 ± 0.12	**3.55 ± 0.05**	2.64 ± 0.07
WT	0.43 ± 0.01	0.50 ± 0.01	0.88 ± 0.02	46.36 ± 0.26	20.58 ± 0.20	2.34 ± 0.07	16.32 ± 0.11	3.84 ± 0.07	2.39 ± 0.13

Values represent the mean ± the standard error of the mean (SEM) of three biological replicates (two technical replicates of each). Bold values correspond to a statistical difference, determined via ANOVA and Dunnett’s post hoc test, *P* < 0.05

### UV–Vis compositional analysis

To estimate the ferulate content of the isolated enzyme lignin, 2–4 mg of enzymatically isolated lignin was dissolved in 3 mL of 1,4-dioxane/water (9:1, v/v). A 3 mL UV–Vis cuvette [1] was charged with 1.9 mL of the 90% dioxane solution and the Shimadzu UV–Vis (UV1900) was baselined from 250 to 900 nm with air as the reference cell. Then 100 µL of the 0.4–1.8 mg/mL lignin solution was added to the cuvette to bring the final lignin concentration to 20–90 µg/mL. The spectra were collected from 900 to 250 nm, making sure the total absorption remained below 1 absorbance unit (Fig. 3, Supplemental Table S3), to remain within the linear region of Beer’s law (*A* = *εCℓ*, *A* = absorption, *ε* = extinction coefficient, *C* = concentration, *ℓ* = path length). The extinction coefficient (*ε*WT as mL∙µg^−1^∙cm^−1^) for poplar WT lignin was determined for *λ *= 900–250 nm using the average *ε* measured for three enzyme-lignin samples from different biological samples with final enzyme lignin concentrations of 50.8, 56.2, and 43.5 µg/mL (Fig. 3, Supplemental Table S3). Note: enzyme lignins are not 100% pure lignin and contain some other cell-wall components such as residual polysaccharides and inorganics (including silica and zirconia) from the ball-milling; the presence of these components artificially decreases the measured lignin extinction coefficients. The extinction coefficients *ε*FA and *εp*HBA (as mL∙µg^−1^∙cm^−1^) were measured using methyl ferulate and methyl *p*-hydroxybenzoate, respectively, Fig. 3.

The estimated amount of ferulate and *p*-hydroxybenzoate present in the enzyme-lignin samples was modelled using a linear combination of *ε*WT_*i*_, *εFA*_*i*_, and *εp*HBA_*i*_, with the constraints that *A*_*i*_ ≥ *d*[*a*(*ε*WT_*i*_) + *b*(*ε*FA_*i*_) + *c*(*εp*HBA_*i*_)] across all wavelengths (i) and *a* + *b* = 1. The values of scaling factors: *a*, *b*, *c*, and *d* were manually determined by iteratively adjusting *a* and *b* to match the A_328_/A_280_ ratio of the lignin sample and *a* and *c* to match the A_260_/A_280_ ratio of the lignin sample. Then the scaling factor *d* was adjusted to minimize the difference between the measured spectra (*A*_obs_) and the model spectra (*A*_model_) keeping [*A*_obs_ – *A*_model_] ≥ 0.00 for all wavelengths. The results of the calculated spectra are overlayed on top of the experimental data in Fig. 3.

### In planta activity screening by UV–Vis compositional analysis

The UV–Vis compositional analysis assay was adapted to rapidly screen transformation events at an early growth stage by using root tissue (leaf or stem would also work) in place of the enzyme lignin. An organosolv lignin was extracted from the root tissues by suspending 10 mg in 1,4-dioxane/methanol and aqueous 2 M HCl (60/30/10, v/v/v) and heating it at 80 °C for 3 h. The extraction solution was cooled to room temperature and filtered, and the UV–Vis spectra were measured from 400 to 250 nm. The ratio of absorption for A_328_/A_280_ provides a fixed value that can be used to quantify the increased amount of ferulate and used to set a threshold for successful transformation. The average ratio for WT poplar is 0.17; here we could apply *a* > 0.20 threshold for a successful transformation event. Note: this selection technique would not select transformations with weak ferulate incorporation into extractable root tissue.

Alternatively, normalization of the data at 280 nm (near *λ*_max_ lignin) allows for direct visual comparison of the results (Fig. 4, Supplemental Table S3). This technique is more forgiving to small changes absorption peak-shape and changes in the sample background.

### Limited-saccharification assay

Hybrid poplar pretreatment and saccharification assays were performed as described [40] with some modifications [18]. Samples of ground xylem tissue [15] were subjected to alkaline pretreatment, with two technical replicates per treatment. Alkaline pretreatment was performed with 62.5 mM NaOH at 90 °C for 3 h. After incubation, the samples were neutralized and washed four times with water. The enzyme cocktail Cellic^®^ CTec3 (Novozymes, Bagsværd, Denmark) was diluted 100 times, and 100 µL was added to each sample. After 4, 24, and 48 h, 20 µL of aliquots were taken from the saccharification sample. The concentration of glucose and xylose in the diluted timepoint samples were determined by Dx-600 anion-exchange HPLC (Dionex, Sunnyvale, CA, USA) as described above.

### Statistical analyses

Statistical analyses were performed using R version 2022.02.3 Build 492 “Prairie Trillium” Release (RStudio PBC., Boston, MA, USA). Dunnett’s post hoc test was conducted using the statistical package DescTools.

## Results

### Generation of transgenic poplar expressing the OsFMT1 gene from rice

Hybrid poplar (*Populus alba x P. grandidentata*; P39) was transformed via *Agrobacterium*-mediated transformation with the *OsFMT1* gene from rice (*LOC_Os05g19910*) previously described by Karlen et al. [32, 33], driven by the lignin-specific *AtC4H* promoter. PCR screening identified several positive transformants in tissue culture and, after propagation of several clones per line, transcript abundance was determined by semi-quantitative PCR to confirm expression. Six *OsFMT1*-transgenic lines were selected and, along with WT lines, were planted in the greenhouse (eight clones per line). The trees were harvested after five months of growth, and relative transcript abundance was determined by RT-qPCR of xylem scrapings. A range in transcript abundance was detected in the transgenic lines, with Line 4 showing the greatest abundance and Line 3 the least, while *OsFMT1* expression was not detected in the WT (Supplemental Figure S1). We present data for the lines in this report based on transcript abundance.

The growth phenotype (diameter and height) of the stem was measured (Supplemental Figure S2). No statistically significant differences in diameter were found between the *OsFMT1* transgenic lines and the WT; however, one line, *OsFMT1* line 7, was significantly shorter than WT.

### Fluorescence microscopy suggests the incorporation of monolignol ferulate conjugates

Phenolic acids and their ester derivatives show an increase in autofluorescence signal after treatment with ammonium hydroxide [41]. We explored whether this trait could be used to visualize/detect the incorporation of ML-FA into the *OsFMT1* poplars. Cross-sections of petioles from the *OsFMT1* transgenic poplars and the positive control after treatment with ammonium hydroxide showed enhanced fluorescence at blue wavelengths, whereas there was no significant base-induced increase in the fluorescent signal for WT and the negative control (Supplemental Figure S3). These findings suggest that we have successfully produced ML-FA *in planta*, and that the conjugates are incorporated into *OsFMT1*-transformed poplar cell walls and, based on the fluorescence around the petiole vascular cells, possibly lignin.

### Higher amounts of *p*-hydroxybenzoate, *p*-coumarate, and ferulate

**Fig. 1:**
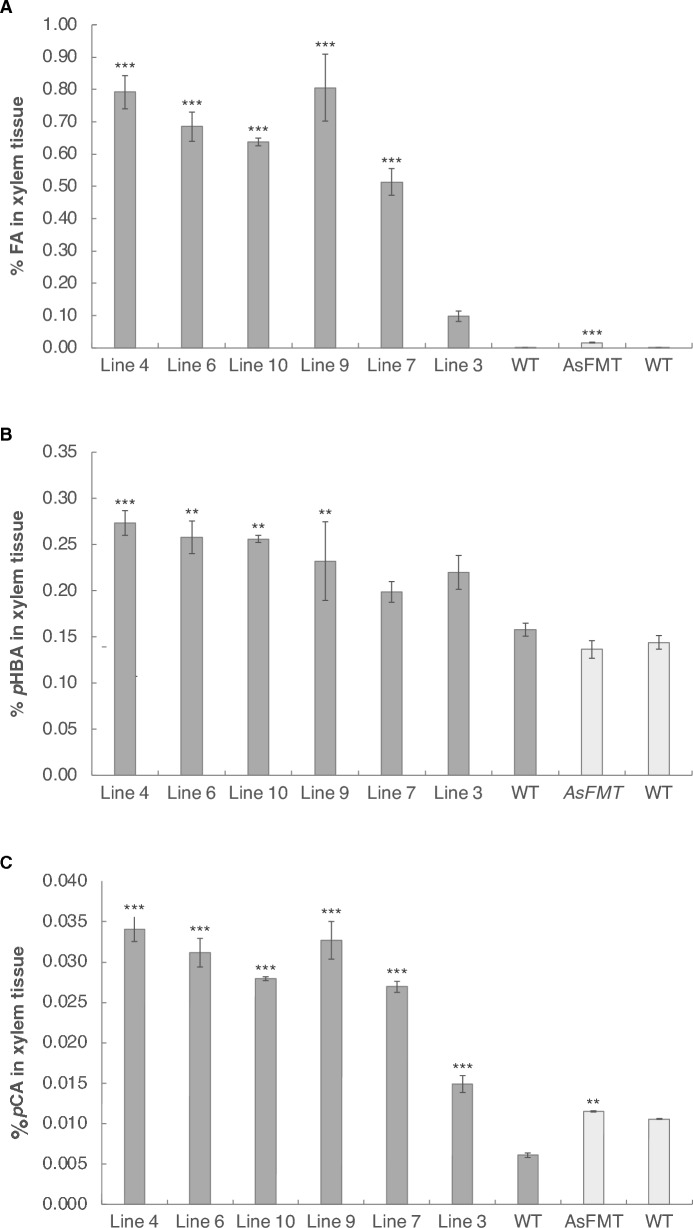
Ester-linked cell-wall bound ferulate, *p*-hydroxybenzoate, and *p*-coumarate determined by alkaline hydrolysis. **A** Amount of ferulate (FA), **B** *p-*hydroxybenzoate (*p*HBA), and **C** *p*-coumarate (*p*CA) in xylem tissue of *OsFMT* and WT poplar trees (dark grey) and in AsFMT (line 7) with its corresponding WT (light grey). *n* = 3 biological replicates for each line (each with two technical replicates), error bars represent SEM. For the *OsFMT* lines compared to WT, statistical differences were determined via ANOVA and Dunnett’s post hoc test, for the AsFMT line compared to its corresponding WT, statistical differences were determined via Student’s *t*-test: *0.05 > *P* > 0.01; **0.01 > *P* > 0.001; and ***P < 0.001

We quantified the amount of ester-linked cell-wall-bound ferulate (FA), *p*HBA, and *p*CA via alkaline hydrolysis (saponification) of extractive-free wood from the greenhouse-grown harvested poplars. HPLC analysis of these samples showed that all the transgenic lines had a significant increase in the amount of ferulate released by alkaline hydrolysis (Fig. 1A), as expected, approaching 1% FA (dry weight in xylem tissue) for Line 4 and Line 9. We also observed an increase of up to 1.7 times in the amount of ester-linked *p*HBA groups in the *OsFMT1* transgenic lines compared to WT (Fig. 1B), as well as an increase of up to 5.5 times in the amount of released *p*CA in the *OsFMT1* transgenic lines compared to WT (Fig. 1C).

To be able to compare our *OsFMT1* poplars with the original *AsFMT* poplars, we performed alkaline hydrolysis on clones from the highest-expressing original transgenic *AsFMT* poplar (*AsFMT* Line 7) and its corresponding WT from Wilkerson et al. [10] (Fig. 1), which were grown in the same greenhouse under the same conditions for a similar duration, but at a different point in time. Although there was a significant increase in the amount of released ferulate after alkaline hydrolysis in the *AsFMT* line compared to the corresponding control trees (11 times higher in *AsFMT* compared to WT), this increase was relatively small compared to the amount of ferulic acid liberated from the *OsFMT1* lines (450 times higher in *OsFMT* compared to WT). Moreover, there was no increase in the amount of *p*HBA released in the *AsFMT* line when compared to its corresponding WT.

**Fig. 2:**
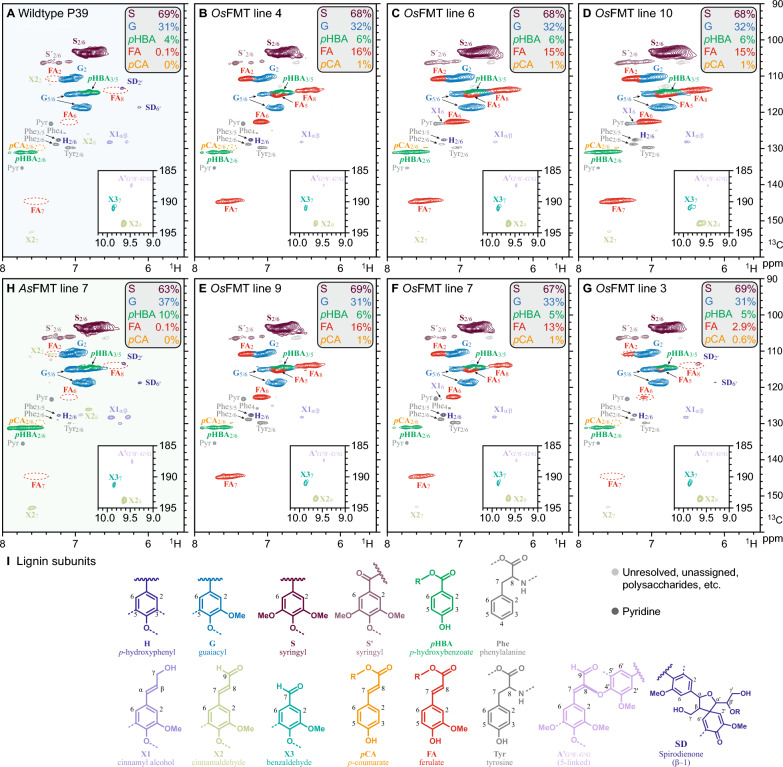
2D-NMR lignin compositional analysis. **A** Heteronuclear single-quantum coherence (HSQC) spectra from a WT poplar. **B–G** HSQC spectra from *OsFMT-*transformed poplar trees. **H** Reference HSQC spectra from the initial *AsFMT*-poplar from the study of Wilkerson et al. 2014. Volume-integrals for G, S, *p*HBA, FA, and *p*CA are given on an S + G = 100% basis (Supplemental Table 1). **I** Substructures colored to correspond to the signals in spectra (**A–H**)

Analysis of enzymatically isolated lignin by 2D heteronuclear single-quantum coherence (HSQC) NMR further supported the data obtained by alkaline hydrolysis and thioacidolysis of the cell wall material, showing an increase in lignin-bound *p*HBA and *p*CA in addition to a significant increase in lignin-bound ferulate in the *OsFMT1* transgenic lines (Fig. 2, Supplemental Table S1). The ferulate signals from the WT and *As*FMT line 7 poplars were too weak to be resolved from the background noise.

Derivatization followed by reductive cleavage (DFRC), an analytical chemical method that cleaves β-ether bonds in lignin polymers while leaving the esters intact [33–35], was performed to quantify the amount of released ML-FA, ML-*p*CA, and ML-*p*HBA specifically in the lignin. We observed an 8–11.5 times increase in the amount of ML-FA for all the transformed lines compared to WT (except Line 3). ML-*p*CA was observed in all the transgenic lines, but was not detected in WT (Table 1, Supplemental Table S2).

### UV–Vis spectroscopy as a new screening method for ferulate and *p*-hydroxybenzoate esters

**Fig. 3:**
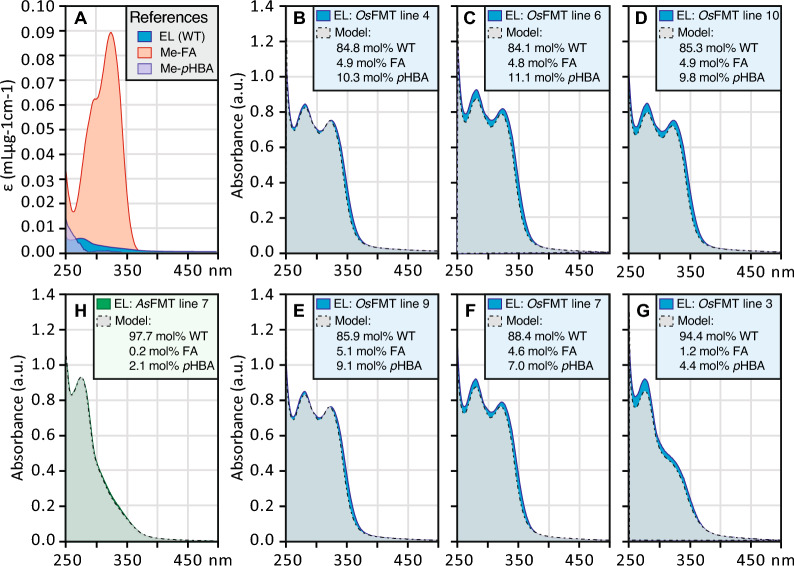
UV–Vis spectra of enzyme lignins (EL) isolated from xylem tissue. **A** Molar extinction coefficients from 250 to 500 nm for wildtype xylem EL (WT), methyl ferulate (Me-FA), and methyl *p*-hydroxybenzoate (Me-*p*HBA). **B–G** UV–Vis spectra of xylem EL isolated from *OsFMT-*transformed poplar trees. **H** UV–Vis spectra of xylem EL isolated from *AsFMT* line 7. Absorption spectra are the average molar extinction spectra (*ε*_*i*_ = Abs_*i*_ / [EL] for *λ*_*i*_ = 250–500) of N = 3 biological replicates normalized to A_250_ = 1. *FA*  ferulate, *pHBA*  *p*-hydroxybenzoate

HSQC 2D NMR is a qualitative tool allowing semi-quantification of the lignin subunits; nevertheless, due to the nature of lignin polymers, lignin end-groups and pendent groups are overestimated [39, 42]. A more quantitative assay may be realized using UV–Vis spectroscopy. We employed Beer’s law (*A*_*i*_ = *ε*_*i*_*Cℓ*, *A*_*i*_ = absorption at *λ*_*i*_, *ε*_*i*_ = extinction coefficient at *λ*_*i*_, *C* = concentration, *ℓ* = pathlength) to model the components of the solutions through a weighted linear combination of components. Assuming that the lignin present in the *OsFMT1*-expressing poplar lines is a linear combination of WT lignin, ferulate, and* p*-hydroxybenzoate esters (pendent groups), then the observed spectrum would be accurately modelled using the linear expression:

1 $$A_{i} = d\left[ {a\left( {\varepsilon {\text{WT}}_{i} } \right) + b\left( {\varepsilon {\text{FA}}_{i} } \right) + c\left( {\varepsilon p{\text{HBA}}_{i} } \right)} \right]$$

where *ε*WT_*i*_, *ε*FA_*i*_, and *εp*HBA_*i*_ are experimentally determined extinction coefficients at *λ*_*i*_ of lignin isolated from WT poplar, ferulate (methyl ferulate), and *p*-hydroxybenzoate (methyl *p*-hydroxybenzoate). The spectral model needs to be constrained to *A*_*i*_ ≥ *d*[*a*(*ε*WT_*i*_) + *b*(*ε*FA_*i*_) + *c*(*εp*HBA_*i*_)] for all *λ*_*i*_, and *a *+ *b* + *c* = 1. These constraints confine the model to be a subset of the observed spectrum and allow for the presence of unassigned components that would be represented as the residual absorption (*A*_R_) as determined by *A*_R_ = *A*_obs_–*A*_model_, based on the constraint in the model, *A*_R_ ≥ 0 for all *λ*_*i*_ wavelengths. Conversion of the relative percentages of a, b, and c to the mol% was performed using the estimated molecular weight of a lignin monomer of 215.7 g/mol, as determined from the weighted average 65:35 S/G of coupled lignin monomers (S–H + OH) = 226.2 g/mol and (G-H + OH) = 196.2 g/mol). The molar percentages for each model were used to calculate the model spectra and included in the associated plot of the observed and simulated UV–Vis spectra, Fig. 3.

The UV–Vis lineshape modelling was consistent with the HSQC NMR data from the lines that highly express *OsFMT1* (lines 5, 6, 7, 9, and 10), suggesting that the lignin is comprised of ~ 4.5–5 mol% ferulate, ~ 7–11 mol% *p*HBA, and ~ 84–88 mol% WT lignin. This is in contrast to the previous best Zip-lignin poplar (*As*FMT-expressing line 7), which was found to be nearly indistinguishable from the corresponding WT samples with a composition of 0.2 mol% ferulate, ~ 2.1 mol% *p*HBA, and ~ 97.7 mol% WT lignin (Fig. 3). It should be noted that this comparison was performed on the same EL material used in the initial Wilkerson et al. [10] study on *As*FMT-poplar. There is a small signal associated with the residual that is redshifted from the modelled spectra which may be due to differences in the extinction coefficients of the methyl esters of FA/*p*HBA and the lignin-bound esters of FA/*p*HBA, and additional minor components like some ferulate coupling products that were not included in the model. The modelled mol% of *p*HBA corresponds to the change from the WT lignin that already contains *p*HBA. The absorption spectrum of *p*HBA is also similar to that of other isolated phenyl groups (e.g., in tyrosine and phenylalanine) and therefore could represent a change in a collection of compounds and not just *p*HBA.

**Fig. 4:**
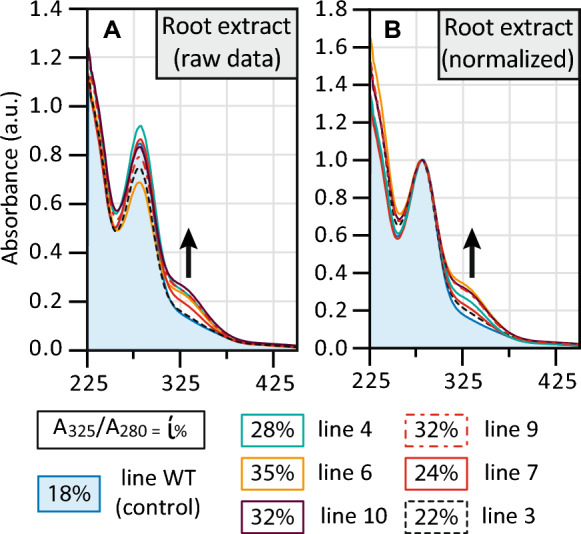
UV–Vis spectra of lignin isolated from the roots of *OsFMT* poplar and WT trees. **A** Raw spectra. **B** Spectra normalized to the 280 nm peak. *N* = 3 biological replicates for each line

We also tested the spectral assay to screen different tissues from the transformation events. Phenotypic screening can be a time-consuming process that typically only reveals the presence of the gene (or protein if fluorescently tagged) and does not provide insight into the in planta activity of the enzyme. In this project, if the FMT enzyme is active and successfully produces ML-FA in planta, then the ML-FA should be incorporated into the cell wall and the resulting lignin would show a second absorption band at 325 nm corresponding to the ferulate. This was demonstrated with lignified primary cell walls and dehydrogenation polymers (DHPs) prepared with 40% coniferyl alcohol and 60% coniferyl ferulate [27]. To test whether the UV–Vis screening assay could be applied to rapidly select the best transformation events, we employed it on root tissue; however, other tissue types (such as stem or leaf) could have been selected. Then to produce a sample suitable for UV–Vis spectroscopy we generated an organosolv lignin using acidic 1,4-dioxane/water because this protocol keeps the lignin subunits mostly intact unlike the Klason lignin or other similar extraction techniques. We then acquired the UV–Vis spectra of the soluble fraction. Successful transformation events were identified when the ratio of root extract absorbance (A_328_/A_280_) was larger than the ratio from WT samples (A_328_/A_280_ = 0.19 ± 0.01, Fig. 4). The highest ratio observed was from line 6 with A_328_/A_280_ = 0.36 ± 0.02. Using a threshold of A_328_/A_280_ > 0.25 would select for lines 4, 6, 7, 9, and 10; the weakly expressing line 3 would be eliminated by this assay. Using such an assay selects transformation events that exhibited positive phenotypical response to the lignin alteration. To confirm the effectiveness of the assays, the screening results were compared to the gene expression. All of the plants with A_328_/A_280_ > 0.20 were identified as successful transformation events.

### Changes in cell wall composition

To analyze the effect of the heterologous gene expression of *OsFMT1* on the cell wall composition in poplar trees, we analyzed structural carbohydrates and used the Klason lignin method to estimate total lignin content. Glucose content was generally increased in the transgenic lines, although not significantly (Table 2). Total lignin content was significantly reduced (up to 13%) in the *OsFMT1* transgenic lines, except for line 3, compared to WT. It should be noted that line 3 also had the lowest transgene abundance, and could effectively act as a surrogate transformation control line (Table 2). Except for the highest expressing line (Line 4), the reduction in total lignin content was largely due to a reduction in the acid-soluble lignin as quantified at 205 nm. Thioacidolysis was also performed on the same woody tissue, and showed a change in the lignin monomer composition. The S/G ratio of thioacidolysis-releasable lignin monomers increased significantly in most transgenic lines compared to WT trees (Table 2).

### Improved enzymatic processing efficiency

**Fig. 5:**
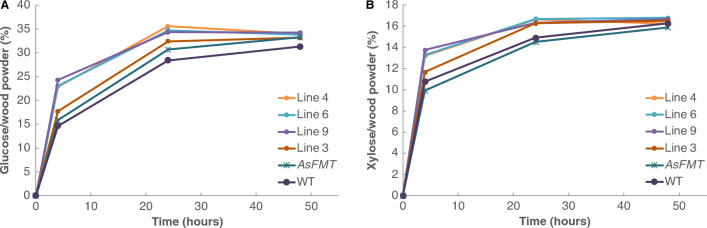
Saccharification efficiency of *OsFMT, AsFMT, and WT* poplar trees. **A** Released glucose, measured after 4 h, 24 h, and 48 h. **B** Released xylose, measured after 4 h, 24 h, and 48 h. *As*FMT = *As*FMT line 7, the highest-expressing line from Wilkerson et al. 2014. Error bars represent SEM. For statistical analysis see Supplemental Figure S4

The increased incorporation of ML-FA observed in the *OsFMT1* poplars results in ester bonds in the lignin polymer, resulting in easier liberation of carbohydrates following alkaline pretreatment and enzymatic hydrolysis. To determine the impact of the increased incorporated ML-FA in the *OsFMT1* poplar trees, we performed a limited-saccharification experiment with alkaline pretreatment (62.5 mM, 90 °C, 3 h). We also added the highest-expressing *AsFMT* poplar (line 7) in this evaluation, to be able to make a comparison between the *OsFMT1* and the *AsFMT* transgenic lines. After 48 h of enzymatic digestion, a saccharification plateau was reached for glucose released (Fig. 5A). We observed a slight, but significant, increase for the *OsFMT1* lines and *AsFMT* line after 48 h of saccharification for the released amount of glucose (Supplemental Figure S4). At the 4 h and 24 h time points, the saccharification efficiency was markedly higher (Supplemental Figure S4), indicating that the saccharification is proceeding at a faster rate, most likely due to increased enzymatic accessibility resulting from cleavage of the incorporated ester bonds in the lignin. A similar trend was observed for the released amount of xylose (Fig. 5B), although there was no significant difference at 48 h (Supplemental Figure S4).

## Discussion

Anthropogenic climate change is having critical impacts on our ecosystems through rising temperatures, increased incidence of drought, salinity elevation, and the spread of pathogens and pests, all affecting plant productivity. Natural systems are concurrently challenged by increasing human pressure: population growth, extended life spans, and continued economic growth. There is therefore an urgent need for a transition from a fossil carbon-based to a bio-based economy including incorporation of fuels and materials derived from lignocellulosic biomass [43–45]. However, lignocellulosic biomass is inherently assembled for strength and resilience, not for industrial processing, and is therefore often referred to as recalcitrant. One of the primary reasons for the recalcitrance is the presence of lignin in the secondary cell wall. For decades, research has focussed on reducing the amount of lignin to improve pulping efficiency and increase cellulose-to-glucose recovery [46]. However, disrupting the phenylpropanoid pathway and monolignol biosynthesis has routinely led to undesirable growth penalties [47–50]. An alternative mechanism to make lignocellulosic biomass more economically attractive for the bio-refinery is to strategically alter the lignin composition and structure by engineering into the lignin polymer non-canonical monomers that create bonds that are easier to cleave and/or that introduce high-value components for downstream chemicals and materials production [5, 7, 51, 52].

This research focused on engineering ester linkages into the lignin polymer and creating a woody substrate more amenable to deconstruction under mild alkaline conditions. The approach has already been successfully demonstrated, creating the so-called Zip-lignin [10, 29]. By incorporating ML-FA ester linkages into the lignin backbone, inherently weaker bonds are introduced into this polymer, creating “Zips”. Non-canonical monomers can also create Zip-lignin. For example, expression of the bacterial *3-dehydroshikimate dehydratase* (*QsuB*) in hybrid poplar not only reduced the total amount of lignin, but probably also resulted in the incorporation of monolignol 3,4-dihydroxybenzoate conjugates into the lignin backbone, creating analogous ester bonds or Zips in the phenolic polymer [24]. The main goal of this research was to increase the abundance of ester-linked ML-FA in lignin with a heterologous *FMT* that is more efficient at producing the required conjugates as driven by a lignin-specific promoter. Here, we used a more metabolically active BAHD acyltransferase, *OsFMT1,* downstream of the *A. thaliana C4H* promoter. The resulting trees exhibited an increase in the amount of FA, *p*HBA, and *p*CA by alkaline hydrolysis, DFRC, and NMR analysis. In addition, a pre-screening microscopic technique showed an increase of phenolic acids in the *OsFMT1* lines, and we successfully demonstrated that a UV–Vis spectrometric method was viable as a screening method to discern the amounts of phenolic acids incorporated into the lignin (Figs. 3 and 4).

Expression of *OsFMT1* in poplar increased the amount of ferulate incorporated into the lignin to levels detectable by 2D HSQC NMR in all six transformed lines examined (Fig. 2). In comparison, the previously reported *AtCESA8*-promoter *As*FMT line 7, with the highest ferulate incorporation [10], did not show NMR-detectable levels of ferulate (Fig. 2H) and required DFRC for more sensitive detection. The new *OsFMT1* lines in this study show a clear signal for ferulate as a pendent group on the lignin via HSQC NMR spectra, as identified through the correlation peaks of the four resolvable ferulate signals (F_2_, F_6_, F_7_, and F_8_, red circles in Fig. 4). If the ferulate moieties of the ML-FA conjugates were covalently bonded into the polymer backbone of the lignin, then either the new aromatic signals associated with the cross-coupled products overlap with the pendent ferulate peaks or the correlation peaks are too weak to identify. The dominant ferulate cross-coupling products are expected to be 4_FA_-O-β_ML_ and 8_FA_-β_ML_ and diferulates from 8 to 5 and 8 to 8 coupling [53]. The large number of possible structures resulting from ferulate coupling [54–56], makes their detection extremely difficult [57]. Previous attempts to incorporate coniferyl ferulate into lignin dehydrogenation polymers (DHP) using maize cell wall suspensions fed coniferyl ferulate along with coniferyl alcohol resulted in detectable levels of coupled and cross-coupled products [53]. In the HSQC NMR of the resulting ferulate/coniferyl alcohol DHP (Fig. 3 in [27]), there are peaks close to FA_2_ (*δ*_H/C_: 7.5/113 ppm) and FA_6_ (*δ*_H/C_: 6.9/123, 6.8/126, 7.4/123, and 7.4/126 ppm) that could be associated with diferulates or cross-coupled structures (Supplemental Figure S5). However, in this study, the lignins isolated from *OsFMT*-expressing poplars did not have obvious signals around FA_2_ and FA_6_ that would indicate the presence of ferulate coupling products. It is therefore probable that, as with the other two common monolignol conjugates (ML-*p*CA and ML-*p*HBA), incorporation of ML-FA occurs primarily through the monolignol with the ferulate moiety functioning as a radical sensitizer for monolignol coupling such that it may not extensively participate directly in the radical coupling reactions of lignification in which radical concentrations, as has long been postulated, are limiting [58, 59]. The xylem tissue assayed in this study was very young and, as the wood matures, it is probable that some of these pendent ferulate units would slowly react with the surrounding lignin to create a more rigid cell wall [60].

Along with the increased amount of ML-FA in the lignin, we also observed an increase in the amount of releasable ML-*p*CA and ML-*p*HBA. The addition of a second acyl donor likely puts added load on the transient pool of monolignols available for coupling. Although we could enhance the (low) amount of ML-FA incorporated into Brachypodium cell walls by reducing competing ML-*p*CA production by downregulating a *p*CA Monolignol Transferase (PMT) [61], expression of *OsPMT* in poplar did not suppress *p*HBMT activity [62]. The transient pool of monolignols in the *OsFMT1*-expressing poplars was sufficient to support both *p*HBMT and FMT activity, in which an increase in ML-*p*HBA content correlated with the expression of the *OsFMT1*. In a cell-free system, the *OsFMT1* enzyme did not exhibit detectable *p*HBMT activity between monolignols and *p*HBA-CoA [63], indicating that the increase in ML-*p*HBA may occur through perturbations in the flux of monolignol or increased expression of *p*HBMT. The *OsFMT1* enzyme exhibits in vitro and in vivo activity with *p*CA-CoA [63], which accounts for the detectable levels of *p*CA on the lignin of poplar lines with high *OsFMT1* expression (lines 4, 9, and 10; Fig. 2A, C, D). The *As*FMT enzyme is much more selective for FA-CoA over *p*CA-CoA; no ML-*p*CA products were detected in an enzyme activity assay when *As*FMT was used, whereas ML-*p*CA products were detected when *Os*FMT1 was used [63]. These observations regarding the various hydroxybenzoates and hydroxycinnamates were supported by the HSQC spectra of the *As*FMT-expressing poplar line 7 (Fig. 2H). Wilkerson et al. 2014 estimated a seven-fold increase in ML-FA incorporated into the lignin in the best *As*FMT poplar line. Based on the saponification of the current best *As*FMT line compared to WT poplars in this study, we suggest a 13-fold increase in the amount of ester-linked ferulate (and no increase in the amount of *p*CA or *p*HBA). In the best *OsFMT1* poplar line we saw a 450-fold increase in ester-linked ferulate. In addition to this change in lignin composition, we also observed a reduced amount of acid-soluble lignin. However, this could also be due to a reduced amount of quantifiable lignin, as the extinction coefficient at 205 nm for ferulate is most likely lower than for other lignin aromatic units, as the UV absorbance of ferulate is higher than for the canonical monolignols [64, 65].

Although NMR spectra may suggest that the majority of ML-FA incorporated into the lignin via monolignol moieties decorates the lignin polymers with pendent esters as for ML-*p*CA and ML-*p*HBA, we still do see an increase in saccharification efficiency, especially at the earlier timepoints. This implies that the modified lignin is easier to break down in an alkaline environment, and permits hydrolysis to proceed at a faster rate. This could be explained by either or both the incorporation of ferulates into the backbone of the polymer (by radical coupling reactions) or by the increased amounts of pendent groups on the lignin polymer (ferulate, *p*CA, and *p*HBA) that render the lignin polymer more difficult to associate with other lignin polymers or other cell wall constituents.

## Conclusions

A decade ago, poplar was transformed with an FMT to increase its degradability [10]. This study demonstrates the benefit of searching for and investigating enzymes with similar functions. These enzymes can have higher efficiency via improved kinetics of the conjugation reaction, or different substrate specificities. When comparing poplars transformed with *AsFMT* and *OsFMT1*, we observed that there was both an increase in the amount of ML-FA incorporated and a broader substrate specificity in the *OsFMT*-transformed poplars. In our case, this broader substrate specificity was beneficial as we also saw an increase in ML-*p*CA and ML-*p*HBA along with the high amounts of ML-FA. The higher amounts of FA, CA, and *p*HBA are of interest as these phenolic acids can be used directly as a commodity chemical output from a lignin-first biorefinery [65–67]. In a lignin-first biorefinery, instead of burning the lignin waste stream, lignin is used for the production of high-value chemicals, improving the economic feasibility of using lignocellulosic biomass as an alternative to fossil fuels [7, 68–71]. *p*HBA can be used to make parabens (used as preservatives in the pharmaceutical and cosmetic industries), terephthalate (a precursor in PET plastics), and acetaminophen [65, 66, 72, 73]. This implies that combining the high catalytic efficiency with the broad substrate efficiency makes *OsFMT* a particularly interesting enzyme to use for both increasing the biomass digestibility and increasing the value for the lignin-first biorefinery. On the other hand, combining high activity with high specificity is still likely the most attractive approach, as separating the different phenolic acids is cumbersome at present [73, 74].

### Supplementary Information

Supplementary Material 1: Methods S1. Generation, selection, and cultivation of transgenic hybrid poplar. Supplemental Figure S1. Expression of *OsFMT* in transformed poplars. Supplemental Figure S2. Height and diameter of *OsFMT* transgenic poplars. Supplemental Figure S3. Autofluorescence microscopy of petiole cross-section of *OsFMT* poplars and controls. Supplemental Figure S4. Saccharification efficiency of *OsFMT, AsFMT, and WT *poplar trees. Supplemental Figure S5. Chemical shifts of model compounds for ferulate coupling products. Supplemental Table S1. The HSQC NMR subunit distribution (given on an S + G = 100% basis) and the major lignin interunit structures. Supplemental Table S2. The quantified DFRC monomers reported for the native lignin monomer and not that of the derivatized substructures. Supplemental Table S3. UV-Vis spectra of preparations of EL (enzyme lignin) for estimations of ferulate content.

## Data Availability

All data generated during this study are included in this published article and its additional file. The raw data is can be made readily available by request to the corresponding author.
