# Supplementary material for: Enhancing monolignol ferulate conjugate levels in poplar lignin via OsFMT1
Source: Biotechnol Biofuels Bioprod. 2024 Jul 13;17:97. doi: 10.1186/s13068-024-02544-y (PMC11246582; doi:10.1186/s13068-024-02544-y)
Supplement: Supplementary file 1 — Supplementary Material 1: Methods S1. Generation, selection, and cultivation of transgenic hybrid poplar. Supplemental Figure S1. Expression of OsFMT in transformed poplars. Supplemental Figure S2. Height and diameter of OsFMT transgenic poplars. Supplemental Figure S3. Autofluorescence microscopy of petiole cross-section of OsFMT poplars and controls. Supplemental Figure S4. Saccharification efficiency of OsFMT, AsFMT, and WT poplar trees. Supplemental Figure S5. Chemical shifts of model compounds for ferulate coupling products. Supplemental Table S1. The HSQC NMR subunit distribution (given on an S + G = 100% basis) and the major lignin interunit structures. Supplemental Table S2. The quantified DFRC monomers reported for the native lignin monomer and not that of the derivatized substructures. Supplemental Table S3. UV-Vis spectra of preparations of EL (enzyme lignin) for estimations of ferulate content. [file 13068_2024_2544_MOESM1_ESM.pdf]

Methods S1. *Generation, selection, and cultivation of transgenic hybrid poplar*

Agrobacterium-mediated transformation was used to generate transgenic hybrid poplar lines expressing a BAHD acyltransferase from rice (*OsFMT*) (GenBank Accession Number LOC_Os05g19910). The *pA6pAtC4H—R1GWR2* backbone was described previously by (Eudes et al., 2015). The gateway cassette was replaced by the *OsFMT* described by Karlen et al., 2016. The expression construct was then transferred into *Agrobacterium tumefaciens* EHA105. Leaf discs of *Populus alba* × *grandidentata* (P39) were harvested from four-week-old tissue culture-grown plants, and ten plates, each containing 50 leaf discs (6 mm^2), were co-cultivated with 30 mL of *Agrobacterium* culture (OD = 0.1–0.2) in 50-mL Falcon tubes for 30 min at 28 °C in a gyratory shaker at 100 rpm. Following co-cultivation, the explants were blotted dry on sterile filter paper and placed abaxial-side-up onto woody plant media (WPM) media containing 0.1 µM 1-naphthaleneacetic acid, 0.1 µM 6-benzylaminopurine and 0.1 µM thidiazuron. The plates were incubated in the dark for two days at room temperature and, after the third day, residual *Agrobacterium* was eliminated by transferring the leaf discs to fresh WPM media as above but also containing 250 mg L^{-1} cefotaxime and 500 mg L^{-1} carbenicillin. The plates were kept in the dark for an additional two days and then explants were transferred to fresh WPM media as above but with 250 mg L^{-1} cefotaxime, 500 mg L^{-1} carbenicillin, and 30 mg L^{-1} hygromycin, and incubated under subdued lighting. After the emergence of shoots, one shoot per leaf disc was excised and placed onto fresh WPM media containing 0.1 µM 6-benzylaminopurine, 250 mg L^{-1} cefotaxime, 500 mg L^{-1} carbenicillin, and 30 mg L^{-1} hygromycin. After 6 weeks of growth under normal lighting (16-h days), explants were transferred to fresh WPM media containing 0.1 µM 1-naphthaleneacetic acid, 250 mg L^{-1} cefotaxime, 500 mg L^{-1} carbenicillin, and 30 mg L^{-1} hygromycin.

PCR-based screening was then used to identify transgenic plant lines. A CTAB-based extraction method was used to isolate DNA from poplar leaves. Transgene incorporation was confirmed by PCR using gene-specific primers: *OsFMT* FW 5´-ATGGTCGCTGTCACCG TGATGAGGA-3´ and *OsFMT* RV 5´-GGCCTTCATCTCATCTTGGAACTCC -3´.

Transgenic trees were propagated and multiplied on antibiotic-free WPM media until six plantlets of each transgenic line as well as non-transformed controls were of similar size. The

trees were then moved to two-gallon pots containing perennial soil (50% peat, 25% fine bark and 25% pumice; pH 6.0), in a greenhouse where they were maintained on flood tables with supplemental lighting (16-h days) and watered daily with fertilized water.

The trees were harvested, the leaves and bark were removed, and the developing xylem was scraped from the stems. Xylem scrapings, bark and leaf tissue were stored at −80 °C, while the remaining stem was left to air dry.

RNA was isolated from developing xylem tissue using the CTAB-based method described by (Kolosova et al., 2004). Contaminating DNA was removed using a DNase I DIGEST kit (Ambion, ThermoFisher Scientific), and 1 µg of DNase-treated RNA was used to generate cDNA with the iScript cDNA synthesis kit (Bio-Rad Labs). The resulting cDNA was stored at -20 °C until use. Real-time quantitative PCR (RT-qPCR) reactions consisted of 10 µL of SsoFast Eva Green Supermix (Bio-Rad Labs), 20 pmol of primers, 1 µL of cDNA, and deionized water to a total volume of 20 µL. qPCR was performed using a CFX 96 System (Bio-Rad Labs) with the following primers: *OsFMT* RT FW2 5´-TTGGAGCAGCACAATTCATC-3´ and *OsFMT* RT RV2 5´-AAAGGACTGGAACGATGGTG-3´. For the housekeeping gene, TIF was used, with the following primers: *TIF* FW: 5´-CTGATAACACAAGTTCCCTGC-3´, and *TIF* RV: 5´-GACGGTATTTTAGCTATGGAATTG-3´. The following thermal cycler regime was used to amplify the 143-bp fragment: 30 s at 95 °C, 39 cycles of 95 °C for 5 s, and 57 °C for 30 s, followed by 95 °C for 30 s, and a melt curve cycle of 55 °C to 95 °C with an increment of 0.5 °C for 5 s.

Autofluorescence microscopy

Petioles from mature leaves of WT and transgenic poplar plants were harvested and persevered in 50% ethanol under refrigeration. Petioles were rehydrated overnight in 100% deionized water and trimmed 2 cm above the stem attachment point. Sections of ~100 µm were prepared with a RapidTome device (Thomas et al., 2023), placed on a slide, treated with a drop of 0.1 M NH_4OH, sealed with a cover slip, and their blue autofluorescence imaged with the 5x objective of a Leica DMI3000 B inverted fluorescence microscope equipped with a standard DAPI filter cube. Identical parameters were used for all images (exposure time 298.5 ms, gain 2.0, saturation 0.75, gamma 0.99). As cell wall ferulate is characteristic of commelinid monocots but typically absent from dicots (Harris and Trethewey, 2010), sections of a stem of the grass teosinte

(*Zea diploperennis*), obtained from the WSU biology greenhouse diversity collection, served as a positive control, and a stem of a dicot, barberry (*Berberis* sp.), served as a negative control.

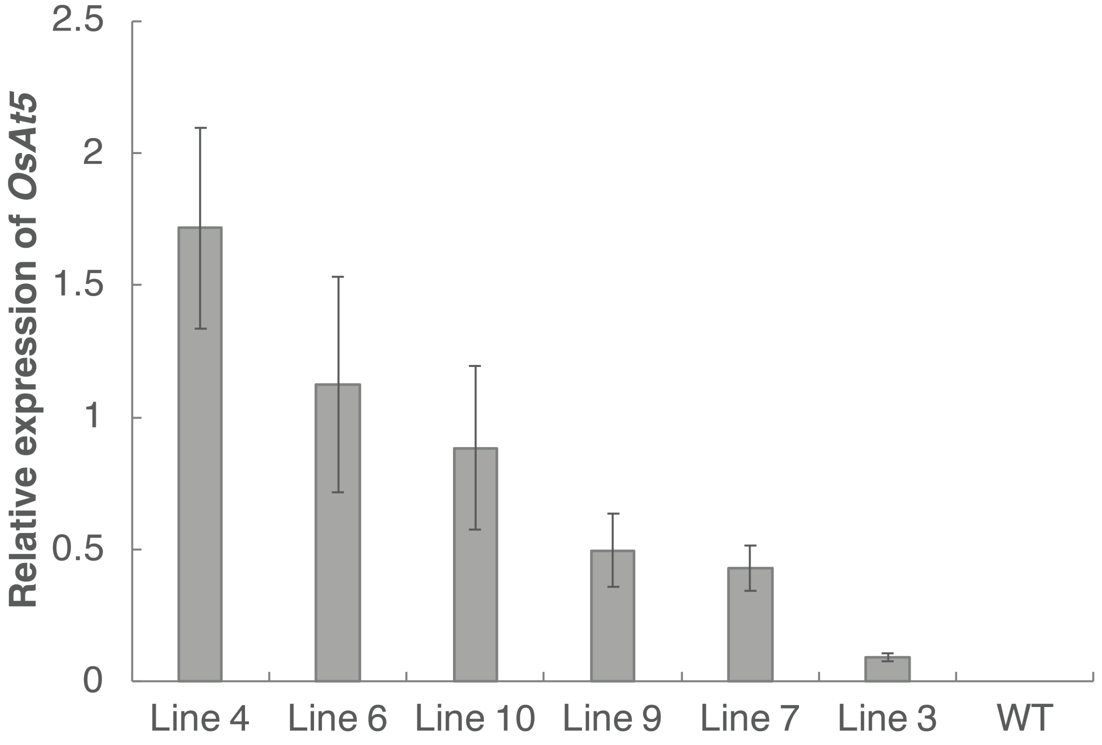

Supplemental Figure S1. Expression of *OsFMT* in transformed poplars. The expression levels of *OsFMT* in xylem tissue are relative to the housekeeping gene *TIF5a*. No expression of *OsFMT* was observed in WT trees. n = 3 biological replicates for each line (each with two or three technical replicates). Error bars represent standard error of the mean (SEM).

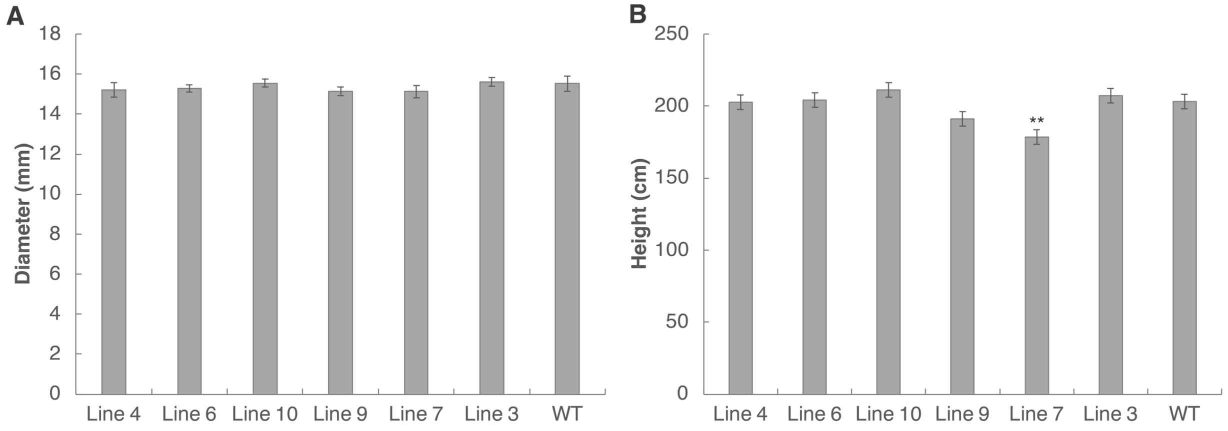

Supplemental Figure S2. Height and diameter of *OsFMT* transgenic poplars. A) Diameter (mm) and B) Height (cm) of *OsFMT* transformed poplars and WT poplars, measured following 5 months of growth in a greenhouse. n = 7 or 8 biological replicates for each line. Error bars represent SEM. Statistical differences were determined via ANOVA and Dunnett's post-hoc test: **0.01 > P > 0.001.

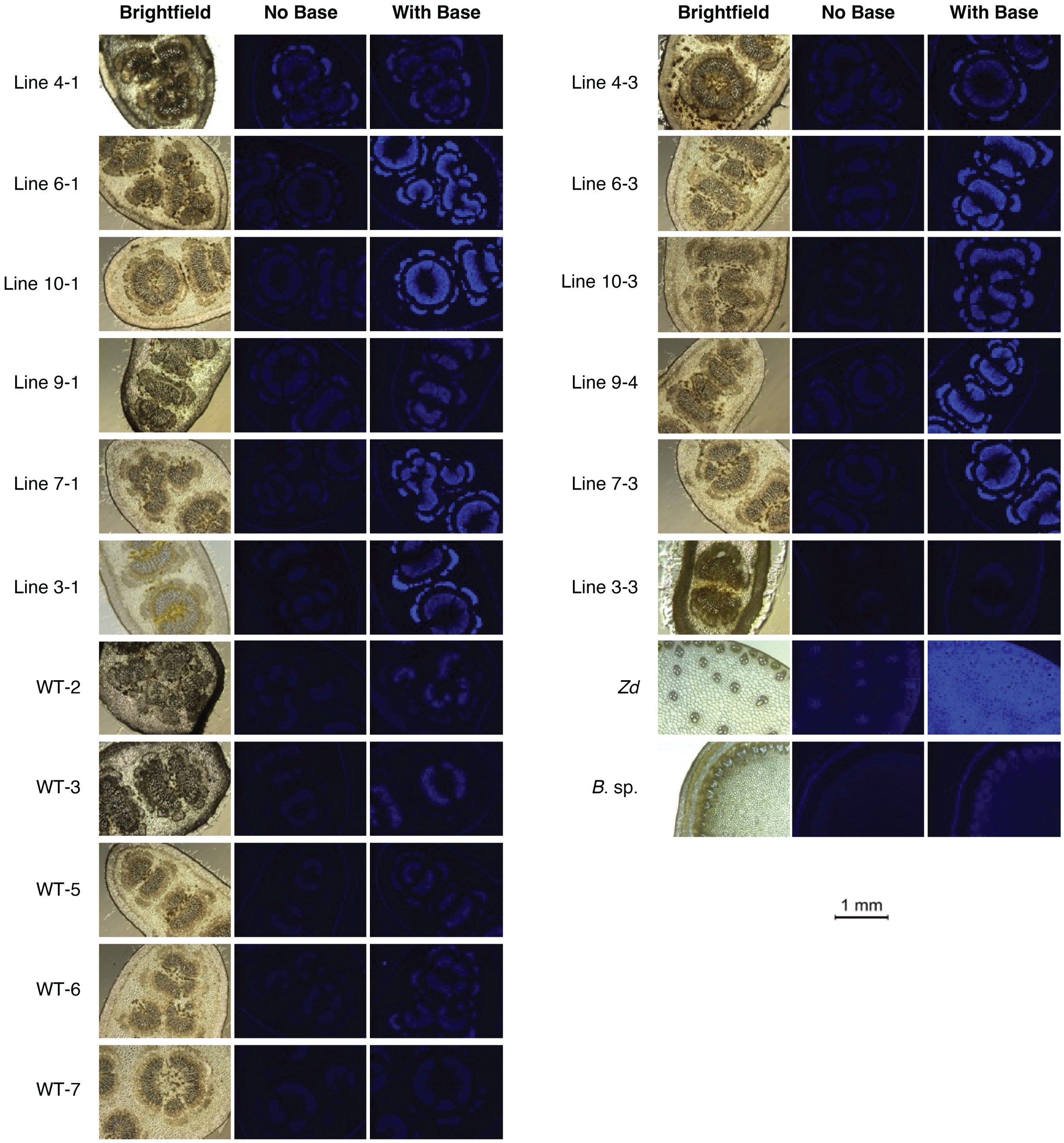

Supplemental Figure S3. Autofluorescence microscopy of petiole cross-section of *OsFMT* poplars and controls. Petioles sections were treated with either water (No Base) or 0.1 M ammonium hydroxide (With Base). Ammonium hydroxide treatment should intensify phenolic acids' autofluorescent signal and that from their ester derivatives. Petiole cross-sections of poplar plants transgenic for *OsFMT* (denoted by numbers) showed greater cell wall autofluorescence with a DAPI filter set than wild-type (WT) plants. The monocot *Zea diploperennis* (*Zd*) is shown as a positive control, and a dicot *Berberis* sp. (*B.* sp.) as a negative control. Magnification 50x, scale bar is 1 mm.

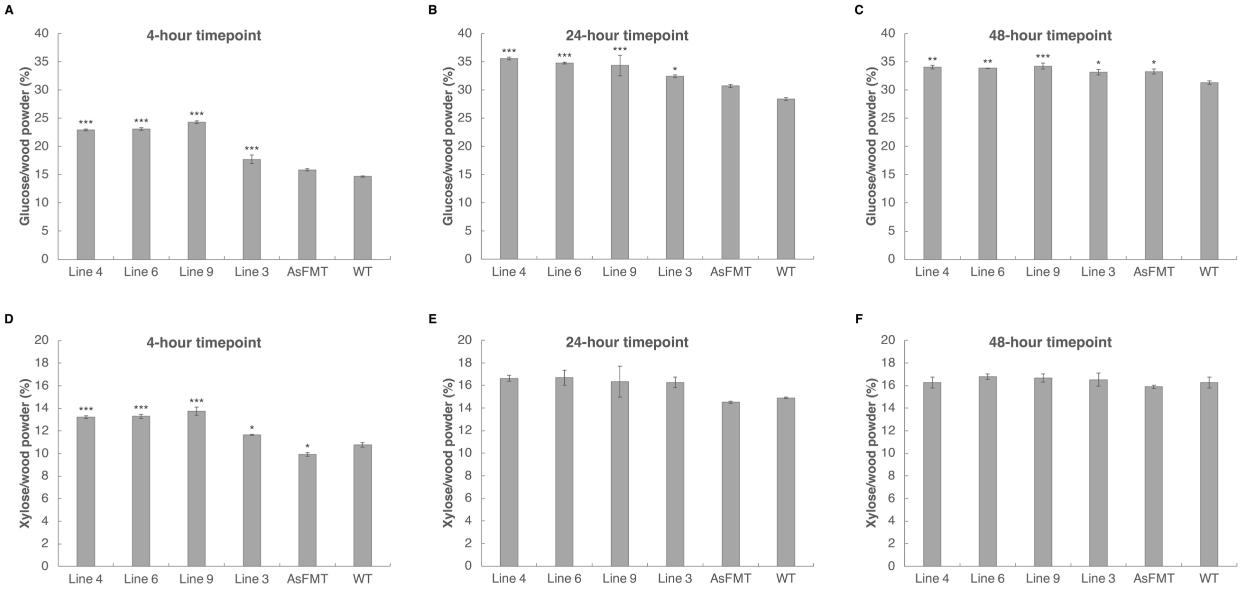

Supplemental Figure S4. Saccharification efficiency of *OsFMT, AsFMT, and WT* poplar trees. A) Released glucose at the 4 h timepoint. B) Released glucose at the 24 h timepoint. C) Released glucose at the 48 h timepoint. D) Released xylose at the 4 h timepoint. E) Released xylose at the 24 h timepoint Released xylose. F) Released xylose at the 48 h timepoint. n = 3 biological replicates for each line (each with two technical replicates). *As*FMT = *As*FMT line 7, the highest-expressing line from Wilkerson et al. 2014. Error bars represent SEM. Statistical differences were determined via ANOVA and Dunnett's post-hoc test: *0.05 > P > 0.01; **0.01 > P > 0.001; and ***P < 0.001.

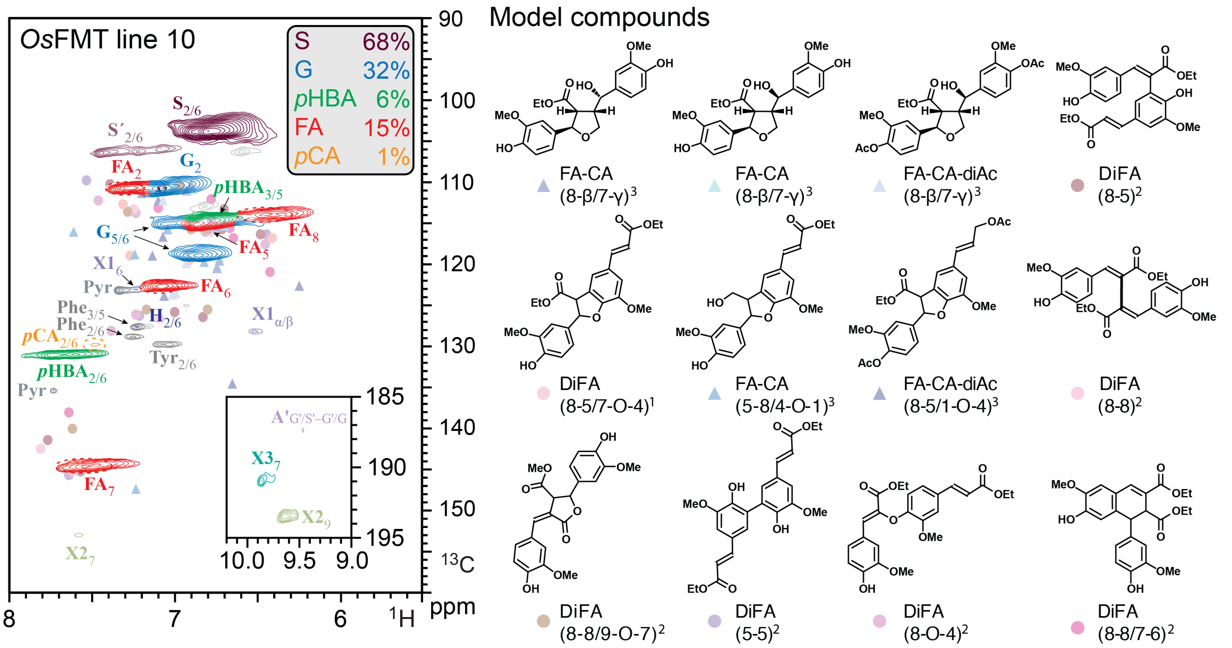

Supplemental Figure S5. Chemical shifts of model compounds for ferulate coupling products. Overlaid onto the HSQC NMR spectrum of lignin isolated from OsFMT-expressing poplar Line 10 are the reported chemical shifts of ferulate-ferulate coupling products (DiFA), ferulate-coniferyl alcohol coupling products (FA-CA), and some acetylated (Ac) derivatives (Ralph et al., 1994, 1992; Zhang et al., 2009).

Supplemental Table S1. The HSQC NMR subunit distribution (given on an S + G = 100% basis) and the major lignin interunit structures [**A**: β-aryl ether (β-O-4), **B**: phenylcoumaran (β-5), and **C**: resinol (β–β) and are given on an **A** + **B** + **C** = 100% basis]. Values are the average of n = 3 biological replicates with the standard error of the mean (SEM). Bold values correspond to a statistical difference, determined via ANOVA and Dunnett's post hoc test, P < 0.05.

	Line 4	Line 6	Line 10	Line 9	Line 7	Line 3	WT
**%S**	68.2 ± 0.8	67.7 ± 0.1	68.0 ± 0.6	69.1 ± <0.0	67.2 ± 0.9	68.9 ± 0.7	69.5 ± 0.1
**%G**	31.8 ± 0.8	32.3 ± 0.1	32.0 ± 0.6	30.9 ± <0.0	32.8 ± 0.9	31.1 ±0.7	30.5 ± 0.1
**%*p*HBA**	**5.7 ± 0.2**	**6.2 ± 0.4**	**6.4 ± 0.1**	5.5 ± 0.4	5.1 ± 0.2	4.9 ± 0.3	4.3 ± 0.3
**%FA**	**16.0 ± 0.6**	**15.3 ± 0.5**	**15.3 ± 0.7**	**15.8 ± 0.5**	**13.1 ± 0.2**	**2.9 ± 0.2**	0.1 ± <0.1
**%*p*CA**	**1.2 ± 0.1**	**1.2 ± <0.1**	**1.0 ± <0.0**	**1.1 ± 0.1**	**0.9 ± 0.1**	**0.6 ± 0.1**	<0.1 ± 0.1
**%CA**	1.2 ± 0.1	1.2 ± <0.1	1.2 ± <0.0	**1.0 ± 0.1**	1.3 ± 0.1	1.5 ± <0.1	1.5 ± 0.1
**%A**	**89.9 ± 0.1**	**89.9 ± 0.1**	**89.5 ± 0.1**	**90.1 ± 0.1**	**89.4 ± 0.5**	**87.9 ± 0.2**	86.7 ± 0.1
**%B**	4.8 ± 0.2	4.8 ± 0.1	5.2 ± 0.1	**4.6 ± 0.1**	5.2 ± 0.3	5.4 ± 0.3	5.6 ± <0.1
**%C**	**5.3 ± 0.1**	**5.3 ± 0.1**	**5.3 ± 0.1**	**5.2 ± <0.1**	**5.4 ± 0.2**	**6.8 ± 0.2**	7.8 ± 0.1

Supplemental Table S2. The quantified DFRC monomers reported for the native lignin monomer and not that of the derivatized substructures. Values are the average of n = 3 biological replicates run in duplicate, with the standard error of the mean (SEM). ND = not detected. Bold values correspond to a statistical difference, determined via ANOVA and Dunnett's post hoc test, P < 0.05. No significant differences were calculated for S_{pCA} and S_{FA}, as these were not detected in WT.

Line #	$H_{OH/OAc}$ mg/g wcw	$G_{OH/OAc}$ mg/g wcw	$S_{OH/OAc}$ mg/g wcw	G_{FA} mg/g wcw	S_{pCA} mg/g wcw	S_{FA} mg/g wcw	S_{pBA} mg/g wcw
**Line 4**	0.1 ± <0.01	**12.8 ± 0.11**	**37.5 ± 0.20**	22.9 ± 0.32	0.3 ± <0.01	4.6 ± <0.04	4.2 ± 0.18
**Line 6**	0.1 ± <0.01	**12.8 ± 0.04**	**38.4 ± 0.14**	**29.1 ± 0.12**	0.4 ± 0.01	4.8 ± 0.02	6.6 ± 1.00
**Line 10**	0.1 ± 0.01	15.5 ± 1.13	**44.4 ± 3.04**	**36.1 ± 1.19**	0.4 ± 0.01	4.4 ± 0.14	7.1 ± 0.66
**Line 9**	0.1 ± <0.01	**12.8 ± 0.11**	**39.0 ± 0.26**	24.2 ± 1.35	0.3 ± 0.01	4.1 ± 0.06	4.0 ± 0.13
**Line 7**	0.1 ± <0.01	14.1 ± 0.08	**39.4 ± 0.17**	19.8 ± 1.05	0.3 ± 0.01	3.7 ± 0.02	3.4 ± 0.15
**Line 3**	0.1 ± <0.01	16.0 ± 0.35	54.5 ± 0.73	5.4 ± 0.30	0.2 ± 0.01	0.8 ± 0.04	4.0 ± 0.21
**WT-Poplar**	0.1 ± <0.01	17.6 ± 0.23	60.2 ± 0.50	3.2 ± 0.10	ND	ND	4.0 ± 0.10

Supplemental Table S3. UV-Vis spectra of preparations of EL (enzyme lignin) for estimations of ferulate content. Values are the average of n = 3 biological replicates run in duplicate, with the standard error of the mean (SEM).

	***Os*FMT Line 4**	***Os*FMT Line 6**	***Os*FMT Line 10**	***Os*FMT Line 9**	***Os*FMT Line 7**	***Os*FMT Line 3**	***As*FMT Line 7**
**Root lignin screening**							
A_{328}/A_{280} (WT:19±1%)	28 ± 2%	35 ± 1%	33 ± 1%	32 ± 2%	24 ± 1%	22 ± 2%	N/A
**EL samples**							
A_{328}/A_{280} (obs)	0.876	0.871	0.873	0.886	0.842	0.485	0.269
A_{328}/A_{280} (sim)	0.876	0.870	0.872	0.885	0.841	0.485	0.269
A_{260}/A_{280} (obs)	0.850	0.860	0.848	0.837	0.832	0.908	0.957
A_{260}/A_{280} (sim)	0.849	0.859	0.847	0.836	0.832	0.908	0.957
**a** (εWT)	87.7%	87.1%	88.0%	88.5%	90.4%	95.7%	98.3%
**b** (εFA)	4.9%	4.8%	4.8%	5.0%	4.6%	1.2%	0.2%
**c** (ε*p*HBA)	7.5%	8.1%	7.1%	6.5%	5.0%	3.2%	1.5%
**d** (scalar)	1.24	1.14	1.20	1.06	0.85	1.30	1.15

References

Eudes, A., Sathitsuksanoh, N., Baidoo, E.E.K., George, A., Liang, Y., Yang, F., Singh, S., Keasling, J.D., Simmons, B.A., Loqué, D., 2015. Expression of a bacterial 3-dehydroshikimate dehydratase reduces lignin content and improves biomass saccharification efficiency. Plant Biotechnol J 13, 1241–1250. https://doi.org/10.1111/pbi.12310

Harris, P.J., Trethewey, J.A.K., 2010. The distribution of ester-linked ferulic acid in the cell walls of angiosperms. Phytochemistry Reviews 9, 19–33. https://doi.org/10.1007/s11101-009-9146-4

Karlen, S.D., Zhang, C., Peck, M.L., Smith, R.A., Padmakshan, D., Helmich, K.E., Free, H.C.A., Lee, S., Smith, B.G., Lu, F., Sedbrook, J.C., Sibout, R., Grabber, J.H., Runge, T.M., Mysore, K.S., Harris, P.J., Bartley, L.E., Ralph, J., 2016. Monolignol ferulate conjugates are naturally incorporated into plant lignins. Sci Adv 2, e1600393. https://doi.org/10.1126/sciadv.1600393

Kolosova, N., Miller, B., Ralph, S., Ellis, B.E., Douglas, C., Ritland, K., Bohlmann, J., 2004. Isolation of high-quality RNA from gymnosperm and angiosperm trees. Biotechniques 36, 821–824. https://doi.org/10.2144/04365ST06

Ralph, J., Helm, R.F., Quideau, S., 1992. Lignin–feruloyl ester cross-links in grasses. Part 2. Model compound syntheses. J Chem Soc Perkin 1 2971–2980. https://doi.org/10.1039/P19920002971

Ralph, J., Quideau, S., Grabber, J.H., Hatfield, R.D., 1994. Identification and synthesis of new ferulic acid dehydrodimers present in grass cell walls. J Chem Soc Perkin 1 3485–3498. https://doi.org/10.1039/P19940003485

Thomas, D.J., Rainbow, J., Bartley, L.E., 2023. The rapid-tome, a 3D-printed microtome, and an updated hand-sectioning method for high-quality plant sectioning. Plant Methods 19, 12. https://doi.org/10.1186/s13007-023-00986-3

Zhang, A., Lu, F., Sun, R., Ralph, J., 2009. Ferulate–coniferyl alcohol cross-coupled products formed by radical coupling reactions. Planta 229, 1099–1108. https://doi.org/10.1007/s00425-009-0894-6
